# Exercise-Induced Release of Pharmacologically Active Substances and Their Relevance for Therapy of Hepatic Injury

**DOI:** 10.3389/fphar.2016.00283

**Published:** 2016-08-30

**Authors:** Hans-Theo Schon, Ralf Weiskirchen

**Affiliations:** Institute of Molecular Pathobiochemistry, Experimental Gene Therapy and Clinical Chemistry, University Hospital RWTH AachenAachen, Germany

**Keywords:** exercise, liver fibrosis, regulatory T cells, cytokines, adaptive immune system, cortisol, monocytes, inflammation

## Abstract

Chronic liver disease (CLD) features constant parenchymal injury and repair together with an increasing hepatic impairment, finally leading to fibrosis and cirrhosis and a heightened risk of hepatocellular carcinoma (HCC). Closely related to the rise in obesity, the worldwide prevalence of nonalcoholic fatty liver disease, the most common form of CLD, has reached an epidemic dimension and is estimated to aﬄict up to 46% of the general population, including more than one out of three U.S. citizens. Up to now there is no effective drug treatment available, which is why recommendations encompass both exercise programs and changes in dietary habits. Exercise is well-known for unleashing potent anti-inflammatory effects, which can principally counteract liver inflammation and chronic low-grade inflammation. This review article summarizes the underlying mechanisms responsible for the exercise-mediated anti-inflammatory effects, illustrates the application in animal models as well as in humans, and highlights the therapeutic value when possible. Based on the available results there is no doubt that exercise can even be beneficial in an advanced stage of liver disease and it is the goal of this review article to provide evidence for the therapeutic impact on fibrosis, cirrhosis, and HCC and to assess whether exercise might be of value as adjuvant therapy in the treatment of CLD. In principle, all exercise programs carried out in these high-risk patients should be guided and observed by qualified healthcare professionals to guarantee the patients’ safety. Nevertheless, it is also necessary to additionally determine the optimal amount and intensity of exercise to maximize its value, which is why further studies are essential.

## Introduction

Chronic liver disease features parenchymal damage and repair, gradually proceeds to fibrosis and finally to cirrhosis, accompanied by increasing hepatic impairment together with a markedly heightened risk for HCC, a pathological process in which cirrhosis and HCC constitute the life-threatening end state (Liu et al., 2013). Due to the widely different etiologies, including chronic infections with hepatotropic viruses, intoxications, especially as a result of alcohol abuse, autoimmune diseases, and metabolic disorder in the context of overweight or obesity, prevention as well as treatment differ depending on the proximate cause (Blachier et al., 2013; Giannitrapani et al., 2013). However, irrespective of the etiology, chronic hepatic inflammation is the response and further stimulates the progress of the disease (Sahasrabuddhe et al., 2012; Seidler et al., 2012; Giannitrapani et al., 2013). Consequently, the intake of NSAIDs for example, indicated a risk reduction of both deaths from CLD and the development of HCC, results that can be ascribed to the ability of NSAIDs to diminish chronic inflammation by blocking prostaglandin synthesis (Sahasrabuddhe et al., 2012) and - specifically regarding aspirin - by down-regulating pro-inflammatory cytokines (Imaeda et al., 2009).

Since controlling the inflammatory state within the liver directly impacts the progression of CLD, the question arises whether this could also be achieved naturally, supplementing standard approaches by inherent properties that are well-known to unleash potent anti-inflammatory effects on their own. Physical activity is principally suitable - beside yielding overall health-promoting benefits - to specifically generate a quantifiable anti-inflammatory activity (e.g., Weinhold et al., 2015) and, in contrast, the degree of physical inactivity commonly occurring on a global scale substantially contributes to chronic diseases and premature death (Booth et al., 2012; Lee et al., 2012). Moreover, the discernible necessity of the body to be active might at least in part be tied to the role that physical activity has played in the survival of our ancestors. Capabilities like running might have served as a tool to successfully search for food, thereby representing a selective advantage and it was hypothesized that endurance running might have been utilized as a hunting method by hominids (Carrier et al., 1984) or to maximize success in scavenging, in either case being of major importance for the evolution of the body shape (Bramble and Lieberman, 2004) and even present-day hunter-gatherers were observed to run after game until it dies from exhaustion (Liebenberg, 2006). Ultimately, both our environment and lifestyle have increasingly changed within a short period of time, whereas our genetic constitution virtually remained the same, creating a conflict that might find its expression in chronic diseases (Eaton et al., 1988), but might likewise be mitigated by reintegrating activities stabilized by evolution, including physical activity, as a permanent feature into our lives.

According to the WHO, physical inactivity is responsible for 6% of deaths worldwide, thus appearing in the fourth position of risk factors for mortality, and consequently, the WHO recommends a specified amount of physical activity to counteract the associated global burden of disease (World Health Organization [WHO], 2010). Additionally, physical activity or more specifically exercise has been included into practice guidelines for the prevention and treatment of NAFLD, a medical condition characterized by a fat mass greater than 5% related to the total liver and the most frequent manifestation of CLD in industrialized nations around the world (Rector and Thyfault, 2011; Neuman et al., 2015; Watanabe et al., 2015; Zhu et al., 2015; Oliveira et al., 2016). These recommendations implicate aerobic exercise of moderate intensity for at least 20 to 60 min on 5 days per week in combination with resistance training performed thrice weekly and for further benefits increasing practice beyond 250 min per week (Oliveira et al., 2016).

The goal of this review is to provide evidence for the therapeutic applicability of exercise in advanced states of CLD, including fibrosis, cirrhosis, and HCC, and to assess whether exercise might be of value as adjuvant therapy in the treatment of CLD.

## The Impacts of Exercise on the Immune System and the Underlying Anti-Inflammatory Mechanisms

**FIGURE 1:**
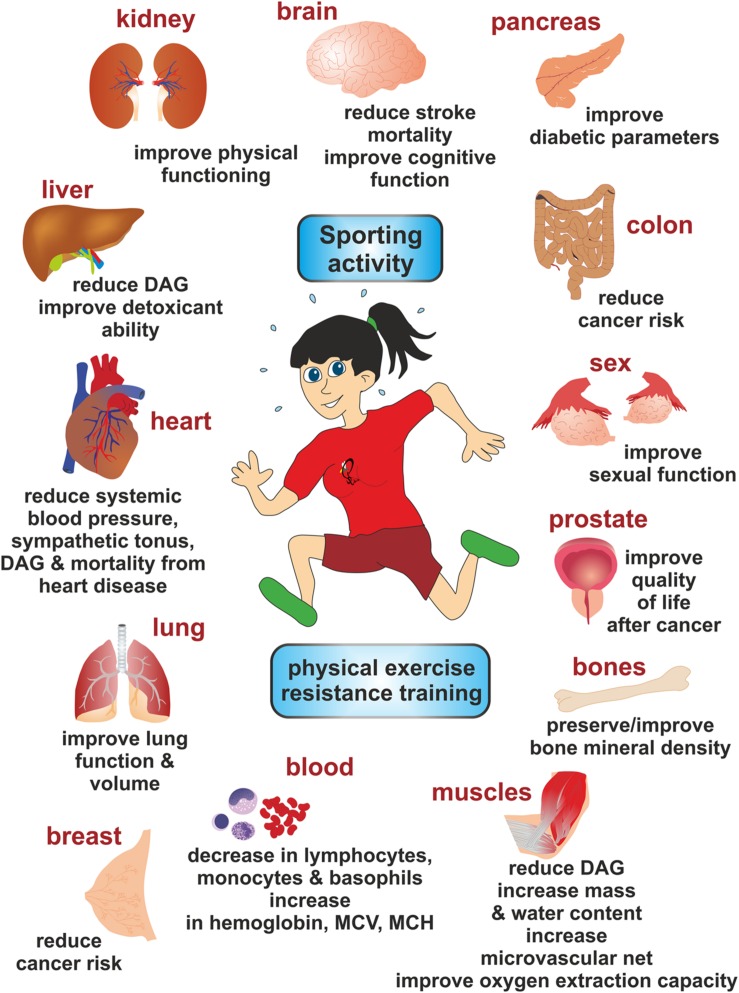
**Representative health-promoting effects of sports, physical exercise, and resistance training on various organs**. Numerous reports have shown that sporting activity promotes health and functionality of muscles, lung, heart, liver, kidney, brain, pancreas, colon, prostate, and bones. In addition, it is reported that doing sports improves sexual function and significantly impacts the composition of the blood. Abbreviations used are: DAG, diacylglyerol; MCH, mean corpuscular hemoglobin; MCV, mean corpuscular volume.

It is obvious that regular endurance exercise promotes favorable structure and metabolism adaptations in contracting organs. However, regular sport, physical exercise, and resistance training have also many health-promoting effects on tissues and organs (**Figure 1**; **Table 1**). Due to the immense technological progress within the last decades more and more people display a predominantly sedentary way of life without the imperative to move adequately. Additionally, dietary habits have changed, processed foods are the standard, and often consuming convenience foods instead of wholesome food further compounds the situation. As a consequence, both physical inactivity and the absence of a healthy and varied diet contribute to a rise in overweight and obesity in the industrialized countries. Of particular importance is the buildup of visceral fat, which attracts pro-inflammatory immune cells, resulting in elevated levels of pro-inflammatory adipokines - factors originating from adipose tissue and unfolding either a pro-inflammatory or an anti-inflammatory action - and the emergence of a low-grade systemic inflammatory condition. This chronic low-grade inflammation is marked by two to threefold elevated systemic concentrations of TNF-α, IL-1, IL-6, IL-1ra, sTNFR, and CRP (Pedersen and Febbraio, 2008). Moreover, it is made responsible for the development of CNCDs, such as atherosclerosis, insulin resistance, and type 2 diabetes (Ouchi et al., 2011), neurodegenerative alterations like depression and dementia (Leonard, 2007), and some cancers (Mathur and Pedersen, 2008). Therefore, it is crucial to reverse this fatal trend by bringing down obesity and low-grade inflammation in order to prevent or ameliorate the associated morbidities. Regular exercise offers a potent instrument to tackle chronic inflammation (Mathur and Pedersen, 2008; Kawanishi et al., 2013b) and its use should not be limited to the prevention of CNCDs, but it can also be useful as a treatment option (Karsten et al., 2016), which is why exercise is meanwhile regarded as medicine (Gleeson et al., 2011). Most of the health-promoting effects of regular exercise are mediated by its anti-inflammatory properties, which have been thoroughly investigated and are now well-founded. In this chapter, we will outline the most relevant anti-inflammatory mechanisms of exercise on the molecular level.

**Table 1:** Health-promoting effects of physical activity/regular exercise.

Health indicator	Health-promoting effects of physical activity/regular exercise	Reference
Mortality and longevity	All-cause mortality is reduced above all by providing cover against atherosclerosis as well as insulin resistance.	Pedersen, 2006
	According to one study with more than 55,000 participants being monitored for a 15-year-period, runners displayed a 30% reduced risk of all-cause mortality and a 45% decreased risk of cardiovascular mortality as compared to non-runners, with a gain in life expectancy of three years. Already slow running for five up to 10 min/day may considerably lower one’s mortality risk.	Lee et al., 2014
Aging	Physical activity protected against the consequences of aging for example by working against the loss of muscle mass together with neuromuscular function.	Cvecka et al., 2015
Cardiovascular disease and hypertension	Cardiovascular disease is marked by chronic systemic inflammation which in turn is counteracted by the anti-inflammatory properties evolved by regular exercise: an increase in anti-inflammatory cytokines and a decrease in the production of TNF-α, thereby protecting against cardiovascular disease and mortality.	Petersen and Pedersen, 2006; Lavie et al., 2015
	Regular aerobic exercise brought about a reduction of blood pressure of 11/5 mm Hg on average.	Börjesson et al., 2016; Heijnen et al., 2016
Diabetes, insulin resistance, obesity, adiposity, and metabolic syndrome	Physical activity reduced the risk of type 2 diabetes. The recommended amount of aerobic exercise as treatment option for type 2 diabetes comprises at least 150 min three times a week in combination with resistance training to build up muscle strength performed at least twice a week.	Mendes et al., 2015


	Metabolic syndrome features abdominal obesity, elevated blood pressure, dyslipidemia, and a disorder of glucose metabolism and pre-diabetes is known as one cause of metabolic syndrome. Within a period of three years the progress from pre-diabetes to diabetes can be lowered by about 58% as a result of lifestyle modifications such as exercise training for losing weight. Recommendations include at least 30 min of moderate training, if possible, every day in combination with additional accommodations of the diet.	Gaesser, 2007; Mayans, 2015
	Obesity is characterized by chronic low-grade inflammation induced by metabolic substances like FFAs whose levels are often heightened in the obese. By binding to pattern recognition receptors including TLRs and FFARs FFAs activate inflammatory signaling pathways responsible for mediating inflammation as well as insulin resistance in both cells displaying metabolic activity and immune cells. By means of exploitation of FFAs, down-regulation of TLR expression and diminishing inflammatory signaling exercise is accordingly capable of restricting chronic low-grade inflammation as well as insulin resistance.	Ringseis et al., 2015
	Exercise leads to an increase in energy consumption and a decrease of (visceral) body fat, but not necessarily to weight loss.	Sallam and Laher, 2016
Osteoporosis	Exercise training can be implemented to prevent and with restrictions even to treat osteoporosis and should comprise various elements, such as resistance training, weight lifting, also balance training, and optionally aerobic units like walking, running, swimming as well as water aerobics. It is recommended to carry out the exercises at least twice and up to four times a week, paying attention to one’s safety.	Moreira et al., 2014; Giangregorio et al., 2015
Cognitive performance	Aerobic exercise, especially running, results in an enhanced release of anandamide, an endocannabinoid, which is responsible for the rise of brain-derived neurotrophic factor (BDNF) levels and the maintenance of heightened BDNF levels after exercise. BDNF-mediated effects on cognitive performance are achieved through stimulation of neurogenesis and synaptic plasticity as well as through improvements concerning learning and memory resulting in a reduced risk of cognitive impairment	Leckie et al., 2014; Heijnen et al., 2016
Mitochondria	Through generation of mitochondria and simultaneous mitophagy to remove damaged mitochondria, the overall function of mitochondria is improved.	Romanello and Sandri, 2016
Neuroplasticity and Neurogenesis	Physical activity promotes neuroplasticity and exercise-mediated neuroplasticity is beneficial in rehabilitation to regain lost motor function after stroke.	Pin-Barre and Laurin, 2015
	Sport enhances neurogenesis. The constant aerobic exercise, for example by using a running wheel, extended adult hippocampal neurogenesis (AHN) in rats, particularly in those that were genetically predisposed to respond to physical training.	Nokia et al., 2016
Cancer	Exercise prevents cancer. In particular the incidence of breast, colorectal, prostate and ovarian cancers is reduced by up to 40% combined with 50 to 60% more cancer survivors, the latter qualifying regular exercise as an important adjuvant therapy. Due to its immunomodulatory properties exercising might be an appropriate strategy to prevent carcinogenesis and neoplastic progression.	Newton and Galvão, 2008; Koelwyn et al., 2015; Champ et al., 2016; Saran et al., 2016
Microbiome	Sports favors diversity of gut bacteria that is conducive to intestinal microbiome as well as the increment of health enhancing bacterial strains.	Cerdá et al., 2016; Cook et al., 2016
Kidney	Regular exercise ameliorates quality of life of patients with chronic kidney disease and aerobic exercise strengthens physical fitness as well as the quality of living in dialysis patients, which is why the authors proposed to include exercise programs into the current treatment concepts of dialysis centers.	Barcellos et al., 2015
Stress, anxiety disorder, depression	Cortisol released in the course of aerobic exercise is subsequently transformed into the inactive cortisone as opposed to cortisol secreted in the wake of chronic psychological stress. Due to this mechanism endurance-trained athletes are better protected against the ramifications of sustained elevated cortisol levels including raised blood pressure, hyperglycemia, and depression. Therefore, sports are thought to reduce the risk of stress-related illnesses like anxiety disorder/depression.	Cerdá et al., 2016; Heijnen et al., 2016

### Regular Exercise Reduces the Amount of Adipose Tissue

**FIGURE 2:**
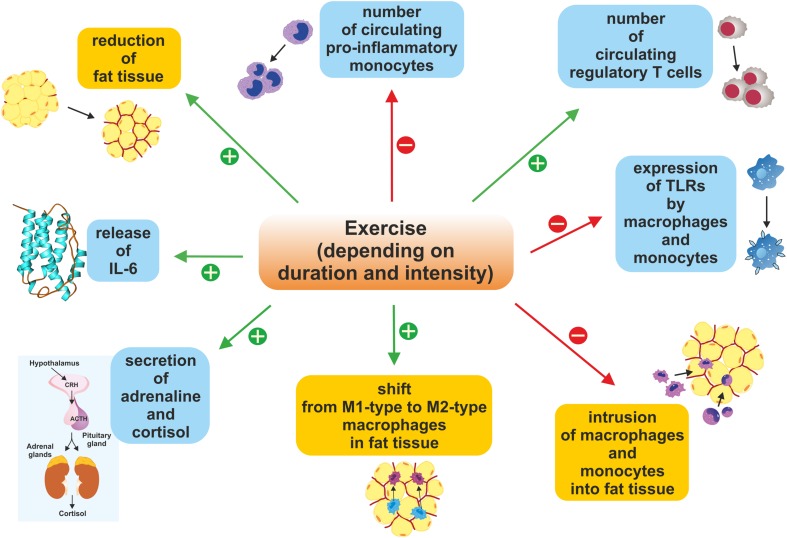
**Duration- and intensity-dependent immunomodulatory changes triggered by exercise and sporting activity**. Exercise increases the number of circulating Tregs, stimulates the secretion of adrenaline and cortisol as well as the release of IL-6, leads to a reduction in fat tissue mass, and impacts the ratio of M1-type to M2-type macrophages in fat tissue. Conversely, sporting activity reduces expression of TLRs, the intrusion of macrophages and monocytes into fat tissue, and also the number of circulating pro-inflammatory monocytes. Altogether, these effects inhibit inflammatory reactions within the body.

The most noticeable outcome of exercise is the loss of body fat over time. Although this primarily seems to affect a person’s shape, the associated immunomodulatory changes are also comprehensive (**Figure 2**). As a matter of fact, abundant fat tissue in obesity is linked to enhanced secretion of pro-inflammatory adipokines including TNF, leptin, IL-6 and IL-18, RBP-4, CCL2, CXCL5, lipocalin 2, and ANGPTL2 (Ouchi et al., 2011). Simultaneously, the production of anti-inflammatory factors, for instance adiponectin and SFRP5, is markedly restricted (Ouchi et al., 2011). This imbalance in favor of pro-inflammatory adipokines finally results in the development of persistent low-grade inflammation.

In contrast, regular physical activity increases energy expenditure and reduces fat depots, thereby reversing the imbalance in pro- and anti-inflammatory adipokines. As a result, circulating levels of the anti-inflammatory factor adiponectin rise and those of the pro-inflammatory adipokines TNF, IL-6, RBP-4, and leptin decrease (Gleeson et al., 2011), in this way contributing to an overall decline of systemic inflammation and in the long run to a stabilization of this condition.

### Active Skeletal Muscle Produces and Releases IL-6

Besides cytokines like IL-8 and IL-15 contracting skeletal muscle also produces and releases IL-6 and collectively these muscle-derived cytokines are also referred to as myokines (Pedersen and Febbraio, 2008). Additionally, IL-6 is also synthesized by various other cell types, for instance, immune cells, adipocytes, fibroblasts, endothelial cells, and different endocrine cells (Fried et al., 1998) and it has numerous functions, but muscle-induced secretion serves the mobilization of both hepatic glucose in order to stabilize blood glucose levels during longer lasting exercise (Steensberg et al., 2000; Pedersen et al., 2001) and fatty acids through lipolysis (Pedersen et al., 2001). Typically, levels of muscle-derived IL-6 are marginal at rest, but in response to acute exercise they can dramatically increase with both duration and intensity and in one study plasma concentration of IL-6 already quadrupled after 30 minutes of running and reached a 25-fold increase after 2.5 h at the end of the training unit, each compared with the value measured before the start of running (Ostrowski et al., 1998). After finishing the marathon distance, the plasma levels of IL-6 were shown to be heightened even 100-fold (Pedersen et al., 2001).

Since intracellular Ca^2+^ leads to a stimulation of IL-6 production in skeletal myocytes, whereas expression of TNF-α - a known activator of IL-6 - is down-regulated at the same time (Keller et al., 2006), skeletal muscle is able to synthesize IL-6 without relying on the pro-inflammatory TNF-α (Nimmo et al., 2013). Accordingly, TNF-α is not produced by skeletal muscle in response to exercise and IL-1β either, both characterized as classic pro-inflammatory cytokines, and moreover IL-6 triggers the production of IL-1ra, IL-10, and sTNFR (Petersen and Pedersen, 2005). Subsequently, these anti-inflammatory factors unfold their specific activity: IL-1ra prevents IL-1 signaling by binding to IL-1 receptors without stimulating a response (Pedersen and Febbraio, 2008) and sTNFR eliminates TNF-α. Finally, IL-10 has been shown to block the synthesis of many pro-inflammatory cytokines, to which IL-1α, IL-1β, and TNF-α belong as well (Petersen and Pedersen, 2005). Compared with the response to acute exercise the production of IL-6 in adipose tissue is triggered by TNF-α and TNF-α-induced IL-6 is considered as pro-inflammatory (Nimmo et al., 2013).

In conclusion, IL-6 can be termed exercise factor by definition, because it is synthesized by skeletal muscle as a result of exercise and is then released into the blood circulation (Catoire and Kersten, 2015).

### Enhanced Secretion of Cortisol and Adrenaline Owing to Exercise Induces a Shift from a Th1 to a Th2 Immune Response

Cortisol, a hormone of the HPA axis, is produced as a result of stress, which can be of a physiological or of psychological nature. A physiological stressor such as exercise feedbacks to the hypothalamus, where CRF and to some extent AVP are synthesized in the PVNs and subsequently released into the hypophyseal vessels. Both factors induce the production and exocytotic release of ACTH in the adrenal gland, which in turn triggers the production of the glucocorticoid cortisol from cholesterol (Stranahan et al., 2008). Furthermore, enhanced circulating levels of IL-6 also raise plasma levels of cortisol *via* ACTH secretion (Steensberg et al., 2003). In addition to preserving blood pressure, the physiological properties of cortisol also include the production of glucose based on proteins and promoting lipometabolism and muscle function (Deuster et al., 1989).

The second major pathway involved in the body’s adaptive response to stress is the SNS, which together with the parasympathetic nervous system and the enteric system constitute the autonomic nervous system. Both the sympathetic and the parasympathetic system have their origin in the brainstem and axons of the SNS pass through the whole body, innervating lymphoid organs as well as the adrenal gland. The adrenal medulla predominantly secretes adrenaline, but also noradrenaline, both at the ratio of about 4:1. Accordingly, the primary products of the SNS are the two catecholamines adrenaline, the principle sympathoadrenal hormone, and noradrenaline, the major sympathetic neurotransmitter (Elenkov et al., 2000).

SNS-induced secretion of adrenaline and noradrenaline starts within seconds and ACTH-mediated release of cortisol within minutes after the beginning of exercise and the circulating levels of cortisol and adrenaline are proportional to the intensity as well as to the duration of activity (Gleeson et al., 2011).

**FIGURE 3:**
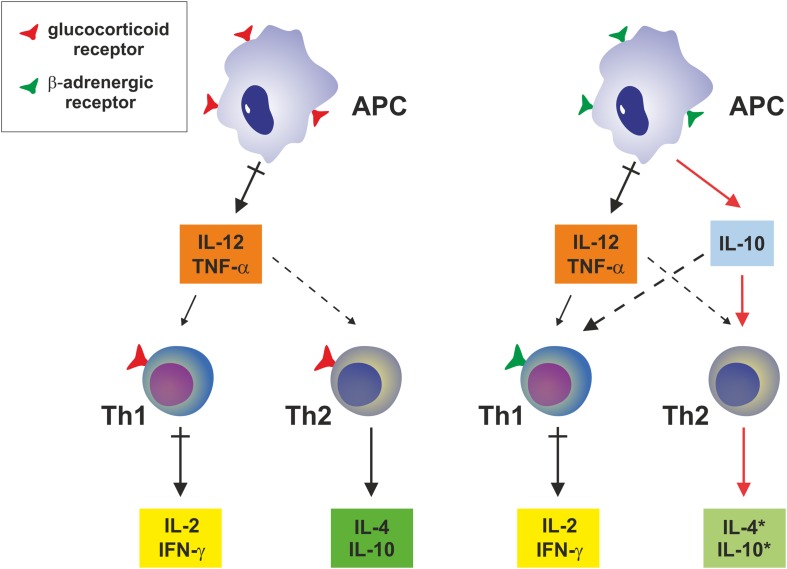
**Systemic effects on expression of pro-inflammatory and anti-inflammatory cytokines by antigen-presenting cells**. Glucocorticoids have no impact on production of IL-10 in monocytes or macrophages, while up-regulating the expression and secretion of IL-10 and IL-4 in Th2 cells. Epinephrine or norepinephrine induces IL-10 production by APCs, while they have no direct impact on Th2 cells. Abbreviations used are. APC, antigen-presenting cell; IFN, interferon; IL, interleukin; Th, T helper cell; TNF-α, tumor necrosis factor-α. This figure was adapted from Elenkov and Chrousos (2002).

Glucocorticoids bind to the glucocorticoid receptor on APCs, such as monocytes and macrophages, block the synthesis of the pro-inflammatory IL-12 and TNF-α (**Figure 3**) and induce a shift from a Th1 cell to a humoral Th2 immune response, accompanied by a decrease in the pro-inflammatory cytokines IFN-γ and IL-2 and an increase in the anti-inflammatory cytokines IL-4 and IL-10, respectively, without influencing IL-10 production directly (Elenkov and Chrousos, 2002). In contrast, catecholamines directly stimulate the synthesis of IL-10 by APCs and also block IL-12 production by these cells, but do not influence Th2 cells immediately. Instead, the reduction in IL-12 enables a Th2-mediated release of the anti-inflammatory acting IL-4 and IL-10 and, additionally, triggers a decrease in the pro-inflammatory cytokines IFN-γ and IL-2 by inhibiting Th1 cells (Elenkov and Chrousos, 2002).

### Exercise Diminishes the Number of Circulating Pro-Inflammatory Monocytes

Monocytes derive from the bone marrow, pass into the blood, and after a few days they end up in different tissues, supplementing the local macrophage pool. Circulating monocytes display a heterogeneous morphology, accounting for roughly 5 to 10% of human leukocytes, and, for instance, in response to pro-inflammatory signals they are attracted to the source and after differentiation they reinforce the body’s defense (Gordon and Taylor, 2005). Heterogeneity of monocytes is tied to different cell surface markers, which allow to distinguish between two subsets: CD14^hi^CD16^-^ cells, also termed classical monocytes, and CD14^+^CD16^+^ cells (Passlick et al., 1989), the latter is characterized as pro-inflammatory due to their higher release of pro-inflammatory cytokines as well as their heightened antigen-presenting ability (Ziegler-Heitbrock, 2007) and their portion of entire monocytes just amounts to 10% (Belge et al., 2002).

In one study, the stimulation with bacterial antigens resulted in a 2.5 times greater expression of the TLR4 on CD14^+^CD16^+^ cells than on CD14^hi^CD16^-^ monocytes (Skinner et al., 2005). TLR4, one of 10 different TLRs in humans, belongs to the pattern-recognition receptors and TLR4 together with CD14 recognizes and binds LPS of gram-negative bacteria, finally leading to a rise in the pro-inflammatory cytokines TNF-α and IL-6, which both play a dominant role in sepsis and other inflammatory diseases (Belge et al., 2002; Schlitt et al., 2004; Skinner et al., 2005).

Notably, there is evidence for an initial increase in pro-inflammatory CD14^+^CD16^+^ monocytes in the blood immediately after an exhausting bout of exercise, followed by a substantial lowering after another hour of rest (Simpson et al., 2009). But it might be difficult to assess whether changes in blood cells are steady or they might occur transiently due to mobilization of these cells and subsequent translocation into distinct tissues (Ziegler-Heitbrock, 2007). Regular exercise unequivocally decreases the portion of pro-inflammatory monocytes in the blood, as it turned out after a twelve-week exercise training of previously untrained individuals, which was able to cut the amount of this monocyte subpopulation by 64% and to induce a drop in TNF-α synthesis (Timmerman et al., 2008).

### Exercise Reduces the Expression of TLRs by Monocytes/Macrophages

Toll-Like receptors are transmembrane receptors, which are able to recognize distinctive, evolutionary conserved patterns exposed on pathogens and to induce both innate and adaptive immune responses (Lancaster et al., 2005). A main feature of the TLR-induced signaling cascade is the activation of APCs, for example monocytes, macrophages, and dendritic cells, which produce pro-inflammatory cytokines together with chemokines and antibacterial substances (Gleeson et al., 2006; Schon and Weiskirchen, 2014), thereby contributing to persistent low-grade inflammation and the formation of chronic diseases (Flynn and McFarlin, 2006; Gleeson et al., 2006).

Results of different studies provide evidence that the expression as well as the function of TLRs is down-regulated in the wake of continual and exhausting exercise. For instance, Lancaster et al. demonstrated that on monocytes the expression of TLR1, TLR2, and TLR4 was distinctly lowered directly after exhausting exercise and also after another two-hour period of rest (Lancaster et al., 2005). Another investigation confirmed these results exclusively for the expression of TLR4 on monocytes: Immediately after one and a half hour of cycling, TLR4 expression declined by 32%, after 1 h at rest by 45%, and after 4 h TLR4 expression reverted to the baseline value, whereas the expression of TLR2 did not change significantly (Oliveira and Gleeson, 2010). And directly after 45 min of running on a treadmill, TLR2 expression decreased by 12% only on pro-inflammatory monocytes as opposed to TLR4 expression, which diminished by 12% after 1 h at rest independently of the subset of the cells (Simpson et al., 2009).

The previous examples underpin the impact of acute exercise on the cell-surface expression of TLRs, so the following study illustrates the effect of chronic exercise on TLR expression, what might carry weight especially in the context of chronic inflammation. After 12 weeks of a combined endurance and resistance training program the cell-surface expression of TLR4 on CD14^+^ cells was significantly lower in both younger and older participants, whereas there was no change in TLR2 expression (Stewart et al., 2005).

Common to the studies above is the fact that they consistently describe the downregulation of the cell-surface expression of TLR4, a result which becomes more important in view of the fact that TLR4 has been shown to play a key role in the activation of pro-inflammatory signaling induced by FFAs in obesity (Jiao et al., 2009).

### Exercise Subdues the Intrusion of Monocytes/Macrophages Into Fat Tissue

In obesity, white adipose tissue serving as energy storage, can be severely augmented. In addition to this increase in mass also changes in both metabolic and endocrine functions occur, resulting among other things in elevated levels of FFAs due to impaired lipolysis (Weisberg et al., 2003; Jiao et al., 2009). Furthermore, it has been demonstrated that these FFAs are responsible for triggering the production of different monocyte chemotactic factors in adipocytes (Nguyen et al., 2007; Jiao et al., 2009), which in turn provoke an accumulation of macrophages in adipose tissue (Kanda et al., 2006; Maffei et al., 2009), especially in the visceral fat depots (Bruun et al., 2005). Finally, these clusters of macrophages within adipose tissue add to the formation of insulin resistance (Kanda et al., 2006).

There are at least two separate pathways whereby FFAs can induce the expression of different monocyte chemotactic factors in the fat cells of the mouse: First, FFAs can upregulate the expression of MCP-1 and MCP-3 at the transcriptional level *via* the JNK pathway (Jiao et al., 2009). Second, FFAs can also trigger the expression of MCP-1, MCP-2, MCP-3, C-C motif chemokine 6 (also known as MRP-1), C-C motif chemokine 9 (also known as MRP-2), and C-C motif chemokine 3 (also known as MIP-1α) *via* NF-κB signaling in both a transcription-dependent and independent manner (Jiao et al., 2009). Notably, only MCP-2 expression remained unaffected by selectively blocking TLR4, suggesting that the expression of this chemokine does not rely on TLR4 (Jiao et al., 2009). In addition, it has been documented that FFA-mediated expression of MCP-1 also depends on JNK signaling in murine macrophages (Nguyen et al., 2007). Beside their function in adipose tissue there is also evidence for a similar role of chemokines and their receptors in attracting monocytes to atherosclerotic plaques (Gautier et al., 2009).

Although the previous reports refer to murine models, the relevance of chemokines for the infiltration of macrophages into adipose tissue of obese humans has also been elicited, since gene expression of both chemokines and their receptors was up-regulated in fat tissue of obese individuals, infiltration of macrophages was elevated, and systemic inflammation concomitantly intensified (Huber et al., 2008). On the other hand the results of several studies employing mice support the notion that exercise training can clearly prevent infiltration of macrophages into adipose tissue, alleviate the associated inflammation (Baynard et al., 2012; Kawanishi et al., 2012; Kawanishi et al., 2013a,c; Linden et al., 2014), and that the suppressed infiltration of macrophages might be related to a decreased expression of chemokines, including MCP-1, MCP-2, and MIP-1α in fat tissue (Kawanishi et al., 2013a).

### Exercise Causes a Shift from M1-Type to M2-Type Macrophages in Fat Tissue

Once attracted to fat tissue, macrophages undergo a phenotypical polarization. Principally, this polarization depends on the microenvironment and results in a change of function, making it possible to largely separate macrophages into two basic groups: M1 or classically activated macrophages as well as M2 or alternatively activated macrophages, which in turn can additionally be subdivided into M2a, M2b, and M2c, relative to the stimuli needed for their activation (Martinez et al., 2008). Cytokines released by Th1 cells, such as INF-γ, and also TLR ligands, including LPS, are able to induce the M1 phenotype, which synthesizes the pro-inflammatory cytokines TNF-α and IL-6 as well as nitric oxide and iNOs (Ouchi et al., 2011). In addition, M1 cells produce IL-12, which plays a central role in the expansion of Th1 cells and the synthesis of INF-γ (Martinez et al., 2008). However, the M2 phenotype occurs upon stimulation with cytokines typical of Th2 cells, such as IL-4 and IL-13, followed by the synthesis of the anti-inflammatory cytokine IL-10, a decrease in the production of pro-inflammatory cytokines, and an upregulation of arginase 1, mannose receptor 1, and IL-1ra gene expression (Ouchi et al., 2011). Similar to the production of IL-12 in the M1-type, M2 cells synthesize IL-10, thereby stimulating the expansion of Th2 cells and the expression of IL-4 and IL-13 by way of an interdependency between Th1/M1 and Th2/M2 cells, respectively (Martinez et al., 2008). Interestingly, polarization of M1 and M2 macrophages can also be reversed in response to the respective Th2 and Th1 cytokines, which might reflect the necessity to be able to either limit or initiate a new immune reaction (Gratchev et al., 2006). Whereas M1 cells display powerful microbicidal characteristics, M2 macrophages are involved in tissue repair and also take part in resolving inflammation (Gordon and Martinez, 2010).

By comparing obese and lean mice, it was observed that in the latter macrophages within the adipose tissue displayed the M2 phenotype, but with advancing adiposity the M1 cells increased in number and finally constituted the predominant macrophage phenotype in the fat tissue of the animals, a condition characterized by enhanced synthesis of TNF-α, IL-6, and nitric oxide altogether leading to inflammation and insulin resistance (Lumeng et al., 2007). Therefore, this study exemplifies how diet may influence the development of an inflammatory state and insulin resistance due to phenotype switching in adipose tissue macrophages.

A direct influence of exercise training on the phenotype of macrophages has been documented by a few studies in rodents. For instance, Kawanishi et al. (2010) found that in the adipose tissue of obese mice mRNA expression of the M1-specific marker CD11c was lowered by chronic exercise as well as that of the M2-specific marker CD163 was considerably heightened and, additionally, that TLR4 expression was distinctly down-regulated at the transcriptional level, leading to the conclusion that chronic exercise might trigger phenotype switching from the M1- to the M2-type and that inflammation might be prevented by means of reduced TLR4 expression. Similar results were obtained from obese rats in which acute exercise also resulted in the M1 to M2 shift and amelioration of insulin signaling in white adipose tissue (Oliveira et al., 2013). Although another investigation of this group had a slightly different focus, it nevertheless documented that treadmill running decreased both the amount of M1-type macrophages in visceral adipose tissue of obese mice and also gene expression of pro-inflammatory cytokines (Kawanishi et al., 2013a).

These examples demonstrate that the anti-inflammatory effects of exercise might at least in part be ascribable to phenotype switching in macrophages and this mechanism has also been described in humans. In order to determine the influence of low-intensity exercise on macrophage differentiation 17 sedentary people joined an exercise program that included a walking distance of 10,000 steps per day, three times a week for a total of eight weeks and blood samples were analyzed before starting the program and after 4 and 8 weeks, respectively. Exercise led to changes in multiple values, including a rise in leukocyte expression of the M2-specific markers CD14, CC motif chemokine 18, also known as AMAC1, and MR, as well as a decline in leukocyte expression of markers characteristic for the M1 phenotype, such as IL-6, TNF-α, and MCP-1 (Yakeu et al., 2010). Concomitantly, circulating levels of IL-10, which is indicative of Th2 cells, were elevated and those of the Th1-specific cytokine IL-6 dropped, collectively indicating that regular low-intensity exercise might favor M2 macrophage polarization (Yakeu et al., 2010).

### Exercise Leads to a Higher Number of Circulating Tregs

The impact of exercise is not restricted to effector cells of the immune system, but there is also an influence on immunomodulatory cells, such as Tregs, which are known for their immunosuppressive activity, since either their lack or a dysfunction causes systemic autoimmunity (Loser and Beissert, 2012) or aggravates inflammation and inflammation-associated injury (Paust et al., 2011). These thymus-derived T cells express the cell-surface markers CD4 and CD25 and, additionally, the transcription factor Foxp3, which is why they are termed CD4^+^ CD25^+^ Foxp3^+^ Tregs (Sakaguchi, 2005). CD25 functions as a receptor for IL-2 (Handzlik et al., 2013), a cytokine necessary for the activation of Tregs by other T cells and that cannot be produced by Tregs on their own (Turka and Walsh, 2008). Furthermore, IL-2 is crucial for the maintenance of Tregs and functionally amplified by TGF-β, an anti-inflammatory cytokine that prevents apoptosis of Tregs throughout their development, thereby also contributing to the immunosuppressive effect of this T cell subset (Schon and Weiskirchen, 2014).

In order to gain insight into the relationship between different T cell subsets in adipose tissue and inflammation combined with insulin resistance in obesity, studies in both rodents and humans were conducted. In mice, it was observed that Tregs were markedly heightened in the abdominal adipose tissue of lean animals, and remarkably diminished in this tissue in their obese, insulin-resistant counterparts, suggesting that Tregs impact tissue inflammation as well as insulin resistance (Feuerer et al., 2009).

With regard to human adipose tissue the distribution of T cells seems to exhibit a different pattern: Whereas the portion of Th1 and cytotoxic T cells remained stable, that of Th2 cells and Tregs in the visceral fat of obese individuals was elevated in comparison to those of the control group (Zeyda et al., 2011). In addition, inflammation related to the rate of Th1 cells and cytotoxic T cells along with Tregs, indicating that, different to the condition in mice, inflammation of adipose tissue in the obese is not caused by a reduction in Tregs (Zeyda et al., 2011).

Concerning the effect of exercise on the number of circulating Tregs, experimental results in mice are in line with those in humans. In a murine study animals were assigned to two different groups, either performing moderate-intensity or high-intensity exercise for a six-week period: High-intensity exercise led to an increment of both Treg percentage and the production of the anti-inflammatory cytokines TGF-β and IL-10, associated with a simultaneous decline in the pro-inflammatory cytokines IL-2 and IFN-γ, whereas moderate-intensity exercise neither affected Treg percentage nor anti-inflammatory cytokines, but up-regulated the expression of pro-inflammatory cytokines instead (Wang et al., 2012). Likewise, Wilson et al. observed an augmentation of circulating CD4^+^ CD25^+^ Tregs as well as of those specifically expressing Foxp3 in response to exercise in young, highly-trained swimmers (Wilson et al., 2009). In order to determine the influence of varying training loads on both circulating Tregs and the synthesis of IL-10 stimulated by antigens in culture, Handzlik et al. compared blood values of participants pertaining to four different groups, each of which corresponding to a distinctive level of physical fitness. The data demonstrated that an elevated number of Tregs as well as enhanced IL-10 synthesis were related to intense workouts and, further, that there was a significant correlation between synthesis of IL-10 and Treg proportion in total lymphocytes (Handzlik et al., 2013).

In a more recent investigation, Weinhold et al. (2015) addressed the question of how much circulating Tregs might be affected by exercise and whether there exists an influence of training loads on the number of Tregs. To that end, the rate of CD3^+^ CD4^+^ CD25^high^ CD127^low^ Tregs was determined in blood samples of 245 elite athletes showing that although there was a rise in Tregs in both male and female athletes, the number of Tregs in females was reduced by an average of 10%, most likely due to an influence of sex-specific hormones. Associated with the boost of Tregs, serum levels of TGF-β likewise raised and, conversely, blocking of TGF-β accelerated T cell growth (Weinhold et al., 2015). These findings yet again underpinned an intensity-dependent impact of endurance exercise on circulating Tregs and, since the effect size was located within the scope of physiological relevance, its association with a pronounced anti-inflammatory status, what on the other hand might contribute to explain the observed phenomenon of overtrained athletes being increasingly prone to infections of the upper respiratory tract (Weinhold et al., 2015).

### Further Mechanisms

In addition to the previously described mechanisms also longer lasting LSS generated by exercise might induce an anti-inflammatory impact on vascular endothelial cells *via* modification of gene expression patterns, including genes known to be implicated in anti-inflammatory as well as in antioxidant processes (Wasserman et al., 2002; Sallam and Laher, 2016). These changes in the phenotype of endothelial cells due to enhanced mechanical force effects might specifically promote vascular health (Wasserman et al., 2002).

Similarly, in recent years there has been increasing evidence for the participation of miRNAs in regulatory processes known to be induced by exercise. Principally, miRNAs are engaged in pro-inflammatory as well as in anti-inflammatory reactions and circulating levels of multiple miRNAs are altered following exercise, in part due to exercise-dependent cell damage, but most of these miRNAs exhibit a mainly anti-inflammatory impact (Makarova et al., 2014). In dendritic cells, for example, oxidized low-density lipoprotein was observed to trigger inflammatory responses during atherosclerosis, a reaction that was subdued by miRNA-181a, resulting in a hampered release of IL-6 and TNF-α and in an increase in the anti-inflammatory IL-10, which in turn had been blocked by oxidized low-density lipoprotein (Wu et al., 2012). And in vascular endothelial cells miRNA-181b was noticed to block inflammatory signaling *via* NF-κB by regulating importin α3, a factor involved in the transport of NF-κB into the nucleus (Sun et al., 2012). In addition, miRNA-181 expression was found to be elevated in response to acute endurance training in murine quadriceps femoris muscles, indicating a possible regulatory function in the adaptive processes to endurance exercise (Safdar et al., 2009) and also rises in circulating levels of miRNA-181b - but not of miRNA-181a - were detected with peaks directly after an uphill course, probably as an immediate consequence of hypoxia (Banzet et al., 2013). All of this suggests that members of the miRNA-181 family might be important for both contributing to an anti-inflammatory environment and inducing adaptations in response to exercise.

Furthermore, characteristic patterns of circulating miRNAs were described, varying from acute exhaustive to sustained aerobic exercise, including miRNAs, like miRNA-146a, which might also be relevant to limit inflammatory reactions (Baggish et al., 2011). Together, these examples illustrate that miRNAs participate in generating the anti-inflammatory effects known to be induced by exercise and one can expect further details in the near future.

Conclusively, a few remarks should be added. Beside the various anti-inflammatory effects it is noteworthy that exercise also induces multiple antioxidant effects, including modulation of redox-sensitive pathways, such as NF-κB and PGC-1α signaling, downregulation of pro-inflammatory signaling associated with lowered generation of RONS, and promoting repair mechanisms involving, for instance, heat shock proteins and telomerase (Sallam and Laher, 2016).

Furthermore, the occurrence of some mechanisms described above is restricted to adipose tissue in obesity, such as the intrusion of macrophages/monocytes into fat tissue as well as phenotype switching in macrophages, whereas others do arise in response to exercise under normal conditions, i.e., in lean individuals, for instance the secretion of cortisol, adrenaline and IL-6 or the rise in the number of circulating Tregs.

Eventually, the relevance of the different anti-inflammatory effects relies at least on the intensity and the duration of a workload. Moreover, it is important whether exercise is performed on a regular basis or just as a single bout. Therefore, the effect induced by catecholamines might play a major role in a short practice of high intensity, whereas IL-6-mediated effects could be supposed following long lasting training and empty glycogen stores and a rise in circulating Tregs might result from enhanced training loads (Gleeson et al., 2011). Performing moderate-intensity training on a regular basis might be a viable conclusion, since this kind of training seems to be suitable for controlling inflammation (Weinhold et al., 2015).

## Significance of Exercise for the Therapy of CLD

In general, patients suffering from CLD are limited in performing extensive physical activities. This is due to the fact that the gradual loss in functionality impacts myriad functions of the liver that physiologically acts as the body’s chemical factory. As such, it is one of the major glycogen storage sites that supply the body with readily available glucose. Liver disease is associated with alterations in processing of glycogen synthesis or breakdown resulting in reduced capacity to provide quick energy during phases of exercise. Chronic ethanol consumption, for example results in a dramatic decrease in liver glycogen concentration that is due to direct effects of ethanol on glycogen metabolism that is exerted at several levels, including posttranslational modulation of enzyme activities (Van Horn et al., 2001). Moreover, the healthy liver is a central storage site for iron and blood, while liver disease is associated with reduced blood oxygenation, intrahepatic angiogenesis and other hemodynamic changes leading to portal hypertension, again preventing extensive exercise. Particularly, this is evident in cirrhotic, high-risk candidates for liver transplantation, in which the oxygen consumption at peak exercise is severely impaired (Dharancy et al., 2008). Similarly, the disease-associated accumulation of toxic products, such as physiological metabolites or xenobiotics (e.g., medication), that results from limited degradation, excretion and detoxification within the diseased liver is a hindrance preventing extensive movements. Moreover, the loss of the hepatic gland function (e.g., production of bile acids) and the decreased anabolic activity in the synthesis of proteins, fats, carbohydrates, and hormones impact the ability to perform intense physical activity. Therefore, in the past, patients with liver disease were often incorrectly advised to restrict their physical activity. The view that CLD is incompatible with physical exercise has dramatically changed during the last years.

Numerous studies in rodents and in humans focused on the influence of exercise on NAFLD, unsurprisingly, since this clinical syndrome occurs with increasing frequency and meanwhile its worldwide prevalence is estimated to be in the range of 4 to 46%, variations mainly depending on the diagnostic procedure being used (Sherif et al., 2016), and aﬄicting more than one out of three US citizens (Noureddin et al., 2015). The prevalence of nonalcoholic steatohepatitis (NASH), the severe subtype of NAFLD, which is marked by liver fat accumulation combined with inflammation for multiple years, is considered varying between 3 to 5% around the world (Sherif et al., 2016). The observed rise in both obesity and the metabolic syndrome, which in turn are closely linked to unwholesome dietary habits, together with an absence of exercise are conducive to NAFLD (Toplak et al., 2016), which has been identified as the leading CLD worldwide (Paradies et al., 2014).

Several experimental studies and clinical observations have shown that regular exercise beneficially impacts the risk of onset and progression of CLD (Berzigotti et al., 2016). Interestingly, under conditions that are associated with an increase of intrahepatic lipid content such as NASH, vigorous activity that allows reducing the overall hepatic lipid content was shown to be more favorable than moderate activity. This was exemplarily demonstrated in a high-fat diet-induced obese mouse model, in which vigorous-intensity and interval treadmill running was more effective in alleviating hepatic steatosis than moderate-intensity and continuous treadmill running (Cho et al., 2015). These results were also confirmed in patients with NAFLD determined by liver biopsy: While vigorous-intensity exercise inversely correlated with NAFLD severity, there was no such effect visible regarding either moderate-intensity training or the total amount of exercise (Kistler et al., 2011). In comparison with NAFLD severity, intensity appears to be of minor importance in terms of NAFLD prevalence. For instance, in more than 5,700 study participants increased amounts of physical activity indicated a decrease in the prevalence of NAFLD, revealing a dose-response relationship between physical activity and NAFLD (Oni et al., 2015). In addition, even a reduction in the time spent sitting together with a rise in physical activity was noticed to result in a lower prevalence of NAFLD in a large number of examined Koreans (Ryu et al., 2015) and also findings of a systematic review pointed at a beneficial impact of a decreased amount of sitting on NAFLD (Whitsett and VanWagner, 2015). Thus, the previous outcomes imply that the therapeutical effect might rather depend on the intensity of exercise, whereas the actual amount of activity might more likely be sufficient for the prevention of NAFLD.

However, also performing ordinary resistance training exclusively composed of pushups and squats three times a week for 12 weeks was accompanied by a gain in muscle mass, a concomitant drop in fat mass, and improved hepatic steatosis as well as traits of the metabolic syndrome in patients suffering from NAFLD (Takahashi et al., 2015).

Also, clinical studies suggested that already short-term exercise is sufficient to reduce hepatocyte apoptosis in obese individuals with NAFLD by improving insulin sensitivity and increasing the oxidative capacity (Fealy et al., 2012). This notion is also confirmed in many other studies showing that active lifestyles and caloric restriction decrease insulin resistance, body weight, and further result in decreased histological signs of liver injury (Gonçalves et al., 2013). Mechanistically, vigorous exercise prevents liver disease through mitochondrial adaptations that affect the content of cytochrome c and the activity of enzymes that are directly linked to fatty acid oxidations (Rector et al., 2011; Gonçalves et al., 2013). Recently, it was shown that the modifying role on lipogenic genes is mediated *via* activation of the AMP-activated protein kinase (AMPK) pathway that is central in the regulation of cellular energy metabolism by modulating the activity of the sterol regulatory element-binding protein family that has been established as a group of transcription factors regulating genes involved in cholesterol and fatty acid synthesis (Cintra et al., 2012).

Interestingly, the exercise-induced antioxidant response in patients who were classified as heavy drinkers was similar to that observed in patients who did not drink heavily, suggesting that most of the observed beneficial effects do not depend on the health status of the liver (Georgakouli et al., 2015). A protective role against hepatic oxidative stress induced by doxorubicin, an antibiotic drug frequently applied to cancer treatment, was reported by researchers who first subjected rats to daily treadmill running for a 6-week period five times per week and subsequently administered the drug: The aerobic training induced a significant rise in the levels of the antioxidant enzymes superoxide dismutase and glutathione peroxidase combined with a simultaneous significant drop in nitric oxide and malondialdehyde, both markers for oxidative stress (Zolfagharzadeh and Roshan, 2013). By extension, it is also conceivable that the hepatoprotective effect of exercise *via* antioxidant mechanisms might similarly contribute to an improved tolerance of drugs by the liver and thus reasonably support drug treatment.

Early studies regarding the effect of lifestyle modification on NAFLD found that a combination of caloric restriction and exercise training led to a reduction in body weight in the range of 5-10% and a significant improvement in or even a normalization of serum ALT levels (Palmer and Schaffner, 1990; Hickman et al., 2002). However, a major problem of these combined approaches was that it could not be clearly determined whether those beneficial effects had to be ascribed predominantly to weight loss or were directly instigated by exercise training. Not until later it has been shown that regular exercise and weight loss independently of one another were able to normalize ALT levels in patients with NAFLD (Suzuki et al., 2005). Subsequently, also other investigations confirmed marked improvements of aminotransferase levels caused by moderate or even low intensity exercise despite no weight loss (Sreenivasa Baba et al., 2006; St. George et al., 2009). Although more recent findings also substantiated these improvements of ALT levels independent of the form of exercise (aerobic *versus* resistance) training and the length of the study (Thompson et al., 2010; Fealy et al., 2012; Khaoshbaten et al., 2013; Shamsoddini et al., 2015), others did not (Johnson et al., 2009; Bacchi et al., 2013).

Similar to the frequently observed decrease in serum levels of ALT, the most diagnostically conclusive biomarker for liver injury, exercise performed on a regular basis has been reported to lower intrahepatic lipid content (Johnson et al., 2012; Loomba and Cortez-Pinto, 2015). A reduction of liver fat in obese individuals solely as a result of regularly performed aerobic exercise without any change in body weight was first documented by Johnson et al. (2009) and the beneficial effect of aerobic exercise training on hepatic fat in the absence of weight loss was then confirmed in several other studies (Finucane et al., 2010; van der Heijden et al., 2010; Sullivan et al., 2012). In addition, also resistance training has been shown to reduce liver fat (e.g., Zelber-Sagi et al., 2014) and there is evidence to suggest that both forms of exercise training are equally effective in decreasing intrahepatic fat independent of weight loss in people aﬄicted with NAFLD (Bacchi et al., 2013; Shamsoddini et al., 2015). Even though ALT levels approximately correlate with the extent of liver inflammation and heightened ALT levels largely match with the severity of NAFLD, there is only a relatively weak correlation between ALT levels and the intensity of hepatic fibrosis (Kim et al., 2008). Due to this, serum ALT levels are not sufficient as an indicator of liver fibrosis on their own, but can be combined with the serum levels of AST, another liver enzyme and also a surrogate marker of hepatic injury, to compute the AST/ALT ratio, which then can be applied to predict advanced fibrosis (Wilkins et al., 2013). Apart from the AST/ALT ratio also the AST/platelet count ratio, the FibroTest (FibroSure) or magnetic resonance elastography are in wide use as noninvasive biomarkers for advanced fibrosis, but liver biopsy constitutes the norm to accurately determine the grade of inflammation as well as the stage of fibrosis or to differentiate between simple hepatic steatosis and NASH (Wilkins et al., 2013). Tissue samples can serve to identify histological hepatic fibrosis for example with the help of staining: Kawanishi et al. (2013c) employed Sirius red and Masson trichrome to display collagen fibers and immunohistochemical staining of α-SMA to specifically flag activated hepatic stellate cells in murine liver tissue, since activation of hepatic stellate cells and excessive deposition of collagen are key events during hepatic fibrogenesis (Schon et al., 2016). Thus, Kawanishi et al. (2013c) succeeded in demonstrating that hepatic fibrosis was alleviated in the exercise group compared with the control group.

Furthermore, some studies suggest that exercise can limit hepatic inflammation and prevent the progression to states of advanced liver damage, such as fibrosis and cirrhosis. In a mutant form of mice, generating a hyperphagic phenotype and fed with atherogenic food, exercise subdued inflammation in adipose tissue and in the liver as well as the development of NASH and of hepatic fibrosis (Haczeyni et al., 2015). Similar results were obtained in another murine study, in which mice were fed with a high-fat diet supplemented with high-fructose water and performed regular exercise training on a treadmill: Likewise hepatic injury, inflammation, and fibrosis decreased in the animals that had undergone training compared with those of the control group (Kawanishi et al., 2012). And reviewing the impact of exercise in humans suffering from NAFLD, Oliveira et al. (2016) stated that exercise is capable of alleviating liver inflammation and oxidative stress by reducing levels of pro-inflammatory cytokines, what may also help to prevent fibrosis, because - beside lipid peroxidation and involvement of Fas ligands - pro-inflammatory cytokines contribute to cumulative liver injury.

Also, exercise has been shown to exert a beneficial influence on accompanying symptoms of NAFLD. For instance, endothelial dysfunction of elastic arteries - a condition that is frequently encountered in patients with NAFLD - indicates a risk for cardiovascular disease and constitutes an early sign of atherosclerosis (Pugh et al., 2014). In marked contrast to measures of conventional care, exercise training of moderate intensity reversed endothelial dysfunction, thereby also lowering the risk for cardiovascular disease characteristic of NAFLD, which is why the authors proposed to prescribe exercise as an essential part of treatment within this risk group (Pugh et al., 2014). The positive effects on endothelial performance did occur without a significant decline in either liver fat or visceral adipose tissue and were thought to be generated by recurrent surges of shear stress acting on endothelial cells (Pugh et al., 2014).

Already almost three decades ago, Ritland (1988) emphasized that individuals suffering from CLD due to viral infection can benefit from regular exercise, because it strengthens oxygen consumption as well as working capacity without any signs of impairing liver function. Even in those in an acute phase of infection, exercise can contribute to maintain physical working capacity, demonstrating that individuals with both acute and CLDs can profit by physical activity (Ritland, 1988). And the notion that exercise in general might be contraindicated during an existing hepatic infection seems to be unfounded as illustrated by the following rather extreme example: From 26 active participants in a 100 km ultra-marathon blood samples were taken one week beforehand, directly after the race, and once again 24 h upon completion and were subsequently analyzed regarding muscle damage, liver function, and oxidative stress parameters. There was a marked increase in all of these markers in the 8 hepatitis B virus carriers as well as in the 18 non-infected runners after the race, but no significant difference was detectable between the two groups themselves (Chiu et al., 2013). Moreover, there was also no indication of virus reactivation in carriers in the 24-h period following the race, altogether leading to the conclusion that neither the risk for inflammation nor for deficits in liver function is greater in hepatitis B virus-infected runners than in their non-infected co-runners upon participation in such a long distance event (Chiu et al., 2013).

Less research has focused on potential benefits derived from exercise in the terminal stage of CLD. This is particularly true for liver cirrhosis, which is known to be associated with a decline in exercise capacity as well as muscle strength and an accelerated start of anaerobic metabolism during practice (Jones et al., 2012). Furthermore, in individuals suffering from portal hypertension, there exist increased risks for hemorrhaging due to heightened portal pressure in response to exercise, a fact which must be balanced by workouts specifically adapted for the needs of these patients (Jones et al., 2012). Up to now there are two studies investigating the effect of exercise on individuals with cirrhosis and both documented exercise-induced enhancements in muscle strength as well as in exercise capacity.

In a study from 1983 five participants with liver biopsy-confirmed cirrhosis were examined at the start of a 12-week practice and then again twice until the end of the program. The maximal oxygen consumption or VO_2max_ - the amount of oxygen, which can maximally be taken up by the body despite an increasing physical effort (Hawkins et al., 2007), and constituting a measure of physical fitness and endurance capacity - initially rose by 19% in the first part and by 29% toward the end of the period without evidence of any complications (Ritland et al., 1983). And in two of four participants in the second study there was an increase in the VO_2peak_, which characterizes the oxygen uptake maximally attained and not necessarily identical with the VO_2max_ (Day et al., 2003), by 21.2 and 27.5%, respectively, and a gain in muscle mass by 20 and 18%, whereas there was no change in the peak oxygen uptake of the other two patients (Campillo et al., 1990). These results admittedly hint at a positive impact of exercise even in patients with cirrhosis, but it is also obvious that further in-depth studies are needed to safely judge the effects in these high-risk patients.

Some recent studies in rodents and in humans also document a beneficial effect of exercise on the development of HCC and on tumor growth. For instance, to determine the influence of regular exercise on the incidence of liver cancer, genetically modified mice were employed that spontaneously develop steatohepatitis as well as HCC. These animals were involved in an exercise program consisting of running on a treadmill during a 32-week period for 1 h a day for 5 days per week. As a result of the exercise program both the quantity and the extent of the tumors within the examined livers were diminished, verifying that exercise is able to restrict tumor cell proliferation (Piguet et al., 2015).

Similar results were obtained in an experimental study with rats, that were split into four groups, one receiving only a low-fat diet, the second a low-fat diet together with swim training, the third a high-fat diet, and the last a high-fat diet combined with swim training (Aguiar e Silva et al., 2012). The swim training, which lasted for eight weeks with five training units per week, led to a reduction in body fat and weight in the two differently fed groups and to ameliorated cholesterol levels in the high-fat diet group, additionally indicating that regular exercise of that kind coupled with a reasonable, i.e., a reduced-fat diet, might be able to abate the development of liver cancer (Aguiar e Silva et al., 2012).

In another clinical study, including 97 patients with HCC, low levels of adiponectin, an adipokine with predominantly anti-inflammatory and anti-diabetic properties, were significantly related to worse histological grades of HCC (Sumie et al., 2011) and generally adiponectin is thought to unfold antitumor effects as well and to assist in offering protection against HCC (Wieser et al., 2012). In addition, adiponectin has been shown to inhibit the progression of HCC *in vitro* by promoting apoptosis *via* thioredoxin, an antioxidant protein (Xing et al., 2015).

Since adiponectin levels can be markedly increased after regular exercise (Markofski et al., 2014), properly dosed exercise should be beneficial for HCC patients. An example for the application of exercise in respective patients is presented by Kaibori et al. (2013), who investigated the impact of exercise on patients with hepatectomy due to HCC. The exercise program started one month prior to surgery, followed by a 1-week pause immediately after the surgical operation and was then resumed for another six months. At the end of this period fat mass as well as whole body mass were significantly reduced associated with a significant rise in peak oxygen consumption and anaerobic threshold (Kaibori et al., 2013).

Finally, according to a recent study a decline in both incidence and tumor growth by more than 60% was demonstrated in response to regular exercise in different mouse models, also including one model for DEN-induced liver cancer (Pedersen et al., 2016). Just 31% of those mice that had received a DEN injection and additionally exercised developed liver tumors as opposed to 75% of the animals in the control group and running also led to a significant decrease in tumor burden (Pedersen et al., 2016). These effects were mediated by NK cells - a crucial cell type of the innate immune system that is able to identify cancer cells without the help of antibodies. After mobilization of NK cells by adrenaline IL-6, which is secreted by muscle cells during exercise, is involved in the further distribution of the immune cells to tumors (Pedersen et al., 2016).

Interestingly, a further study reported the activation of NK cells induced by one bout of exercise. At least for 24 h after execution of a half marathon, both histone acetylation and the expression of a functional marker of NK cells remained elevated, suggesting that exercise might be helpful to reduce cancer risk as well as recurrence rates (Zimmer et al., 2015).

Based on the previous examples, exercise might even be of worth for advanced stages of CLD. However, from the clinical perspective, the beneficial effects of exercise have not been translated into positive outcomes and there is an urgent need to implement strategies to promote a behavior change, including the implementation of daily resistance training-based exercise regimens to reduce liver fat, and preventing and treatment of NAFLD (Johnson et al., 2012; Loomba and Cortez-Pinto, 2015). Personalized exercise programs dependent on the severity of hepatic disease might also be beneficial to enhance blood oxygenation, improve energy metabolism, reduce disease-associated atrophies, or to increase the patient’s mood by stimulating the release of endorphins and modulating neurotransmitter synthesis or activity.

## Concluding Remarks

Despite constant efforts to develop effective drugs, up to now there exist only a few agents, which are applicable to the medication of CLD. Therefore, the conventional approach focuses on prevention and early diagnosis, whereas at an advanced stage the only remaining possibility often consists in liver transplantation. Independent of the etiology, there is always an underlying chronic inflammation in the heart of CLD and dampening the inflammatory state can slow down the progression of the disease, thereby preserving and ameliorating liver function.

Exercise has repeatedly been shown to unleash potent anti-inflammatory effects and, conversely, a lack of physical activity is believed to be responsible for a wide spectrum of chronic diseases. Increased amounts of physical activity and exercise meanwhile constitute a classic in the prevention and treatment of diseases of the cardiovascular system, such as coronary heart disease or heart failure (Lavie et al., 2015). Therefore, it is plausible reasoning to make use of these benefits in the context of CLD, too, while keeping the patients’ safety in mind. There is no doubt that exercise can even be beneficial in an advanced stage of liver disease and - under the guidance of qualified healthcare professionals - can be an appropriate adjuvant therapy. In response to exercise, various endogenous agents are produced, which unfold a quantifiable pharmacological activity, thereby contributing to recovery. Since exercise has been shown to be effective in both prevention and therapy of certain chronic diseases, consequently the notion of exercise as medicine was coined (Gleeson et al., 2011). But it is also necessary to determine the optimal amount and intensity of exercise and to identify the most suitable workout to maximize its value. In order to obtain this knowledge, further studies are essential. As long as these questions are unanswered, it will yet be better to be physically active for at least 5 to 10 min every day than to stay inactive, since such small amounts are already connected with a clear drop in the risks of all-cause and cardiovascular mortality (Lee et al., 2014).

## Author Contributions

H-TS and RW have written this review and agree to be accountable for the content of the work.

## Conflict of Interest Statement

The authors declare that the research was conducted in the absence of any commercial or financial relationships that could be construed as a potential conflict of interest.

## ABBREVIATIONS

α-SMA: α-smooth muscle actin
ACTH: adrenocorticotropin hormone
ALT: alanine aminotransferase
AMP: adenosine monophosphate
AMPK: AMP-activated protein kinase
ANGPTL2: angiopoietin-like protein 2
APC: antigen-presenting cell
AST: aspartate aminotransferase
AVP: arginine vasopressin
CCL2: CC-chemokine ligand 2
CNCDs: chronic non-communicable disease(s)
CLD: chronic liver disease
CRF: corticotropin releasing factor
CRP: C-reactive protein
CXCL5: CXC-chemokine ligand 5
DEN: diethylnitrosamine
FFA: free fatty acid
Foxp3: transcription factor forkhead box P3
HCC: hepatocellular carcinoma
HPA: hypothalamic-pituitary-adrenal
IL-1: interleukin-1
IL-1ra: IL-1 receptor antagonist
iNOs: inducible nitric oxide synthase
JNK: c-Jun NH2-terminal kinase
LPS: lipopolysaccharides
LSS: laminar shear stress
MCP-1: monocyte chemoattractant protein-1
miRNA: microRNA
NAFLD: non-alcoholic fatty liver disease
NASH: non-alcoholic steatohepatitis
NF-κB: nuclear factor-κB
NSAIDs: non-steroidal anti-inflammatory drugs
PGC: peroxisome proliferator-activated receptor gamma co-activator
PVNs: paraventricular neurons
RBP-4: retinol-binding protein-4
RONS: reactive oxygen and nitrogen species
SFRP5: secreted frizzled-related protein 5
SNS: sympathetic nervous system
sTNF-R: soluble TNF-α-receptor
TGF: transforming growth factor
Th: T helper
TLR: Toll-like receptor
TNF: tumor necrosis factor
Tregs: regulatory T cells
WHO: World Health Organization.
